# The impact of the oxytocin receptor gene (OXTR) on facial affect recognition in psychosis

**DOI:** 10.1192/j.eurpsy.2022.518

**Published:** 2022-09-01

**Authors:** V. Mikhailova, M. Alfimova, T. Lezheiko, M. Gabaeva, V. Plakunova, V. Golimbet

**Affiliations:** Mental Health Research Center, Clinical Genetics Laboratory, Moscow, Russian Federation

**Keywords:** oxytocin receptor gene, social emotions, schizophrénia, Psychosis

## Abstract

**Introduction:**

Oxytocin is considered as potential treatment targeting social dysfunctions in psychoses. However, results of clinical trials are inconsistent which may be due to genetic variation in the oxytocin system involved in social information processing.

**Objectives:**

To examine the effect of the *OXTR* polymorphism and its interaction with childhood adversity (CA) on facial affect recognition (FAR) in psychotic patients.

**Methods:**

Patients with schizophrenic and affective psychotic disorders (n=934) completed a task that required labeling six basic and three social emotions. The polymorphisms rs53576 and rs7632287 within the *OXTR* locus were genotyped and dichotomized based on prior research. For 65% of the sample, information on CA defined as parental alcoholism or psychiatric illness was collected. The polymorphisms’ role in FAR was assessed using ANCOVAs adjusted for sex, age, and diagnosis.

**Results:**

After Bonferroni correction, there was a significant effect of rs53576, mainly driven by the difference between genotypes in the affective patients. GG-homozygotes recognized emotions better than A-allele carriers. A nominally significant effect in the expected direction was also found for rs7632287. CA influenced FAR but did not interact with any genotype.

**Conclusions:**

The results provide further evidence that *OXTR* impacts social cognition and behavior in diverse cohorts, including psychotic patients, with rs53576 GG-homozygotes having enhanced social competencies. However, we have failed to confirm that *OXTR* modulates the relations between CA and FAR in psychosis. The difference in FAR between genotypes was more pronounced in affective patients, which might be due to more severe FAR deficits in schizophrenia.

**Disclosure:**

No significant relationships.

